# Mediterranean Dietary Pattern in Type 2 Diabetes Management: Pathways and Clinical Evidence

**DOI:** 10.3390/biomedicines14061350

**Published:** 2026-06-15

**Authors:** Dubravka Majić Milotić, Tomislav Bulum, Kristijan Peroš

**Affiliations:** 1 Department of Diabetes and Endocrinology, Vuk Vrhovac University Clinic for Diabetes, Endocrinology and Metabolic Diseases, Merkur University Hospital, 10000 Zagreb, Croatia; 2 School of Medicine, University of Zagreb, 10000 Zagreb, Croatia

**Keywords:** Mediterranean diet, type 2 diabetes, plant-based foods, healthy fats, omega-3 fatty acids

## Abstract

The Mediterranean diet (MedDiet) has emerged as a promising dietary strategy for the prevention and management of type 2 diabetes mellitus (T2DM). This narrative review provides a comprehensive synthesis linking the biological pathways of the MedDiet with established clinical evidence. Adherence to this traditional dietary pattern—characterized by a high intake of fiber, complex carbohydrates, antioxidants, and healthy fats—has demonstrated significant benefits in terms of glycemic control, enhanced insulin sensitivity, and overall metabolic health. Mechanistically, the review explains how the MedDiet improves health by modulating key physiological processes, including anti-inflammatory and antioxidant pathways, the regulation of branched-chain amino acid metabolism, the enhancement of short-chain fatty acid production via gut microbiota modulation, and upregulated incretin effects. Importantly, this review explains how the MedDiet complements modern medications, including glucagon-like peptide-1 (GLP-1) receptor agonists and sodium–glucose cotransporter-2 (SGLT-2) inhibitors. By integrating molecular mechanisms with human clinical outcomes, this narrative review addresses multiple aspects of the MedDiet in both the prevention and management of T2DM including glycemic control, weight management, and cardiovascular risk reduction, rendering it a valuable dietary strategy for both the prevention and treatment of this chronic condition.

## 1. Introduction

**Figure 1:**
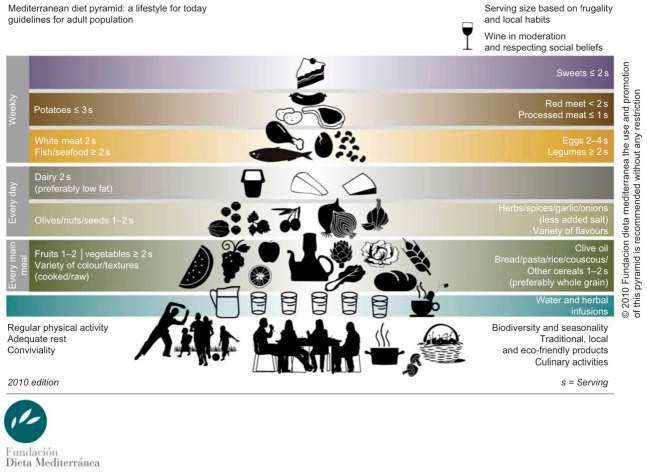
The Mediterranean Diet Pyramid [3].

The Mediterranean diet (MedDiet) represents a well-established and extensively investigated dietary pattern characterized by a high intake of plant-based foods and a favorable lipid profile. This nutritional model emphasizes the consumption of vegetables, fruits, whole grains, legumes, nuts, and extra-virgin olive oil (EVOO) as the primary source of dietary fat, alongside moderate consumption of fish—particularly rich in omega-3 fatty acids—and dairy products such as yogurt and cheese [1,2]. In contrast, the intake of red and processed meats, refined carbohydrates, sweets, and sugar-sweetened beverages is minimized, while moderate wine consumption during meals is traditionally permitted (Figure 1) [1,2]. Beyond its compositional characteristics, the MedDiet encompasses broader lifestyle components, including the use of fresh, seasonal, and locally sourced ingredients, culinary simplicity, and the promotion of social interaction during meals.

The scientific basis for the MedDiet originates from the seminal work of Ancel Keys and the Seven Countries Study, which established a strong association between dietary patterns and cardiovascular health outcomes across diverse populations [4]. This pioneering research demonstrated that populations adhering to traditional MedDiet patterns exhibited significantly lower rates of coronary artery disease compared with those in Northern Europe and the United States. Over subsequent decades, a growing body of epidemiological and interventional research has confirmed the beneficial effects of the MedDiet across a wide spectrum of chronic diseases, including type 2 diabetes mellitus (T2DM). The global prevalence of T2DM has reached epidemic proportions, posing a substantial public health challenge. The clinical burden of T2DM extends beyond glycemic dysregulation and includes both microvascular and macrovascular complications [5,6,7,8,9,10,11,12,13].

Current clinical guidelines for T2DM management advocate a comprehensive, patient-centered approach that integrates pharmacological therapy with lifestyle modification [14]. Advances in pharmacotherapy, including the introduction of glucagon-like peptide-1 (GLP-1) receptor agonists, sodium–glucose cotransporter-2 (SGLT2) inhibitors, and dipeptidyl peptidase-4 (DPP-4) inhibitors, have significantly improved glycemic control and cardiovascular outcomes. Nevertheless, lifestyle interventions, particularly dietary modifications, remain a cornerstone of both the prevention and management of T2DM, given their cost-effectiveness and broad impact on metabolic health. Within this framework, the MedDiet has emerged as a highly effective dietary strategy for the prevention and management of T2DM. Numerous studies have demonstrated that adherence to the MedDiet is associated with improved glycemic control, enhanced insulin sensitivity, and favorable effects on body weight and lipid metabolism [3,15,16,17,18,19,20,21,22,23,24,25]. Prospective cohort studies and randomized controlled trials have consistently demonstrated that adherence to the MedDiet is associated with a reduced risk of incident T2DM [26]. Notably, large-scale intervention studies such as the Finnish Diabetes Prevention Study and the Diabetes Prevention Program have shown that lifestyle interventions incorporating dietary modification and increased physical activity can reduce the incidence of T2DM by approximately 58% among individuals with impaired glucose tolerance [27,28].

Despite the robust evidence supporting the MedDiet, further research is warranted to elucidate the precise molecular mechanisms underlying its protective effects and to explore its interaction with emerging pharmacological therapies. A better understanding of how dietary and pharmacological interventions can be integrated may facilitate the development of more effective and personalized strategies for T2DM management. This narrative review addressed multiple aspects of the MedDiet in both the prevention and management of T2DM, including glycemic control, weight management, and cardiovascular risk reduction, rendering it a valuable dietary strategy for both the prevention and treatment of this chronic condition.

## 2. Materials and Methods

An online literature search was conducted to cover the period from the publication of the PREDIMED (Prevención con Dieta Mediterránea) study to January 2026. The databases searched were PubMed and Google Scholar. Search strategies combined key terms related to the MedDiet, T2DM, diabetes prevention, diabetes incidence, glycemic control, prediabetes, dietary patterns, cardiovascular risk factors, weight management, and insulin resistance. Boolean operators and synonyms were used to maximize the retrieval of relevant literature. An example of a representative PubMed search string used in our literature search is as follows: (“Mediterranean diet” OR “MedDiet” OR “Mediterranean dietary pattern”) AND (“Type 2 diabetes” OR “T2DM” OR “Diabetes prevention” OR “Diabetes incidence” OR “Glycemic control” OR “HbA1c”) AND (“randomized controlled trial” OR “systematic review” OR “meta-analysis” OR “prospective cohort studies”). The same search strings were adapted for Google Scholar, using quotation marks for exact phrases and Boolean operators (AND, OR) to combine terms. Additionally, a manual search of the reference lists of the included articles and relevant reviews was conducted to identify further pertinent studies. Two independent reviewers screened the titles, abstracts, and full texts to ensure consistency and minimize selection bias.

The inclusion criteria were as follows: (1) studies assessing the effects of the MedDiet on the incidence of T2DM, glycemic control in T2DM, and cardiovascular risk factors in adult populations; (2) randomized controlled trials, systematic reviews, meta-analyses, and prospective cohort studies with human subjects published in peer-reviewed journals; (3) reporting diabetes incidence or quantitative clinical outcomes such as HbA1c, fasting glucose, lipid profiles, blood pressure, and body mass index; and (4) published in English during the searched period. The exclusion criteria were as follows: (1) studies focusing on type 1 diabetes, gestational diabetes, or pediatric populations; (2) studies lacking clear dietary pattern definitions or interventions; (3) non-peer-reviewed articles, editorials, commentaries, or conference abstracts without full data; and (4) studies not reporting relevant clinical outcomes related to T2DM management or prevention. The screening process involved removing duplicates after the initial search, followed by independent title and abstract screening against the inclusion and exclusion criteria. The full texts of potentially eligible studies were reviewed for final inclusion. Data extraction focuses on study design, population characteristics, dietary intervention or pattern, and clinical outcomes relevant to the incidence of T2DM, glycemic control, and cardiovascular risk factors. Our review encompassed data from hundreds of thousands of participants included in systematic reviews, meta-analyses, randomized clinical trials, and prospective cohort studies. Having established the search methodology, this review now proceeds to a comprehensive analysis of seminal research that has substantially contributed to the current understanding of the influence of the MedDiet on the prevention and management of T2DM.

## 3. Evidence Supporting the Role of the Mediterranean Diet in the Prevention of Type 2 Diabetes

Building upon the foundational understanding of the role of the MedDiet, this section examines specific studies that elucidate its efficacy in T2DM prevention. Martinez-Gonzalez et al. conducted a comprehensive evaluation of dietary patterns using an extensive food questionnaire comprising 136 items [29]. They employed the scoring system developed by Trichopoulou et al. to quantify adherence to the MedDiet [30]. This methodology facilitates the assessment of dietary adherence and provides clear insights into associated health benefits. The scoring system developed by Trichopoulou et al. assigns numerical values based on the consumption of beneficial food groups and moderation in less healthy ones, providing a quantifiable measure of adherence to the MedDiet dietary pattern. It evaluates nine dietary components. These included fat balance, alcohol moderation, consumption of specific food groups (legumes, grains, vegetables, fruits, nuts, and fish), low meat consumption, and moderate dairy intake. Researchers calculated the consumption of each food group, including fruits, vegetables, cereals, legumes, fish, meat, fast foods, and dairy products, by summing all the items consumed within those categories. The questionnaire also investigated fat and oil usage, cooking techniques, and dietary supplements [29,31]. Each component was scored as ‘1’ for adequate daily consumption and ‘0’ for inadequate intake. Through the application of this scoring system to assess dietary adherence, researchers have provided compelling evidence regarding the role of diet in mitigating T2DM risk. Utilizing this scoring methodology, Martinez-Gonzalez et al. demonstrated significant health advantages associated with the MedDiet, indicating a substantial 83% reduction in T2DM risk among individuals with high adherence (score greater than six) [29]. The considerable reduction in T2DM risk identified by Martinez-Gonzalez et al. underscores the potential of the MedDiet as a fundamental component of public health strategies for the prevention of diabetes. The primary limitation of this research was the small number of newly diagnosed diabetes cases, which may have compromised the statistical power of the study. An additional limitation pertains to the quality of the nutritional assessment because dietary self-reporting is prone to bias and inaccuracies, which can significantly impact the reliability of the findings. Therefore, more objective measures of dietary adherence, such as biomarkers of nutrient intake, are required in future research.

**Figure 2:**
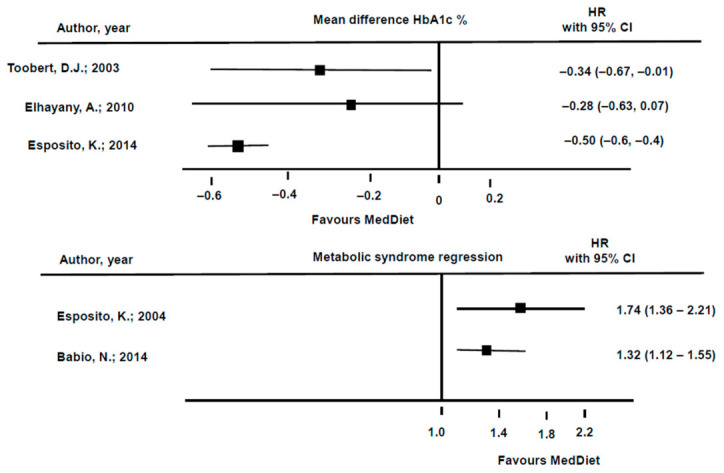
A meta-analysis was conducted on three long-term randomized controlled trials (RCTs) examining the impact of the Mediterranean diet on glycemic control in diabetes, as depicted in the top panel [34,35,36]. The two arms of the RCT conducted by Elhayany et al. [35] were combined for analysis. Additionally, a meta-analysis of the regression of metabolic syndrome through the Mediterranean diet is presented in the bottom panel [37,38]. The two arms in the RCT by Babio et al. [38] were preliminarily combined for this analysis (HbA1c, glycosylated hemoglobin; MedDiet, Mediterranean diet).

After the seminal work of Martinez-Gonzalez et al., further studies have corroborated the connection between the MedDiet and a reduced risk of T2DM, thereby enhancing our understanding of its preventive potential. Expanding on these findings, ongoing scientific inquiry, including a systematic meta-analysis of ten prospective studies, aimed to elucidate the potential protective mechanisms of the MedDiet dietary pattern in preventing diabetes onset. An extensive meta-analysis incorporating data from ten long-term studies revealed a substantial 23% reduction in T2DM risk in individuals who consistently followed a MedDiet [32]. This analysis underscored the enduring protective effects of diet, as evidenced by studies with follow-up periods exceeding 10 years. This investigation had several limitations, including heterogeneous methodologies for assessing adherence to the MedDiet, variations in controlling for confounding variables, variable durations of follow-up, and differences in the number of diabetes cases observed. These limitations may have influenced the overall research outcomes. The compelling nature of these findings has catalyzed further comparative investigations, as exemplified by Esposito’s comprehensive systematic review [33]. This review assessed the comparative efficacy of the MedDiet against control diets in T2DM management. Esposito’s systematic examination of meta-analyses and randomized controlled trials (RCTs) yielded significant results: individuals following the MedDiet experienced a 15–80% decrease in metabolic syndrome risk, a 49% enhanced likelihood of metabolic syndrome remission over a 2–5-year period, and a 19–23% reduction in future diabetes incidence relative to control diets in T2DM and pre-diabetes treatment. Moreover, a meta-analysis of extended clinical trials demonstrated that following the MedDiet resulted in a notable reduction in HbA1c levels (−0.47%), indicative of enhanced glycemic control in T2DM. This finding further substantiates the superiority of the MedDiet over conventional care or low-fat dietary interventions (Figure 2, top panel).

The PREDIMED study was conducted in Spain as a multicenter trial to investigate the impact of the MedDiet on the prevention of cardiovascular disease. Participants were randomly distributed among three nutritional protocols: a MedDiet enhanced with EVOO, a MedDiet supplemented with a variety of nuts, or a low-fat diet, which served as the control group [39]. Although the primary focus of the PREDIMED study was on cardiovascular outcomes, following a median follow-up duration of 4.0 years, a subgroup analysis identified diabetes incidence rates of 10.1% (95% CI 5.1–15.1), 11.0% (5.9–16.1), and 17.9% (11.4–24.4) in the EVOO, nut, and control groups, respectively [37]. The adjusted hazard ratios for developing T2DM were calculated as 0.47 (0.23–0.97) for the EVOO cohort and 0.47 (0.23–0.98) for the nut cohort, in comparison to the control cohort [40]. When both MedDiet groups were combined, the hazard ratio for diabetes was 0.47 (95% CI 0.26–0.87) compared to the control group, and a higher level of adherence to the MedDiet was inversely correlated with the incidence of T2DM across all study arms [40]. The incidence of T2DM was also reduced in patients with high cardiovascular risk following a MedDiet enriched with EVOO [41]. Notably, the risk of T2DM diminished even in the absence of significant alterations in body weight or physical activity levels. This study has several limitations. The study predominantly focused on an older Mediterranean population with an elevated risk of cardiovascular conditions, raising concerns regarding the generalizability of its findings to healthier or younger populations in other regions. Moreover, the lifestyle score utilized to assess the relationship between changes in dietary objectives and diabetes incidence may not have encompassed the full spectrum of dietary alterations, potentially limiting the detection of significant differences between the interventions. The absence of oral glucose tolerance tests (OGTTs) for some participants limited potential diabetes diagnoses to cases with blood glucose levels of 7.0 mmol/L or higher in the fasting state, with confirmation provided by a follow-up test. This limitation may have led to an artificially lower overall incidence rate. Additionally, the study’s findings are further limited by the relatively small sample size, which included fewer than 55 incident cases.

The updated systematic review and dose–response meta-analysis of adherence to the MedDiet and the risk of T2DM synthesized evidence from 24 prospective cohort studies and one RCT, encompassing nearly one million participants (991,878) and over 68,000 incident T2DM cases, with an average follow-up of 12.2 years. The authors concluded that higher adherence to the MedDiet is associated with a moderate-certainty, significant dose-dependent reduction in the risk of developing T2DM. Specifically, each 2-point increase in adherence corresponded to an 8% lower risk of developing T2DM. This association was consistent across various subgroups and was supported by evidence from a randomized controlled trial [42]. The main limitations were that the studies included in the network meta-analysis were mainly of very low to moderate quality, partly due to the lack of allocation concealment and blinding, heterogeneity in the outcome of HbA1c, and the included studies did not describe whether participants actually followed the dietary approach in the protocol design or collect data on the daily intake of each dietary component, thus potentially influencing the actual effect on outcome indicators. In addition, the number of long-term studies on glycemic control in T2DM patients was limited, and the findings need to be considered in light of the very low to moderate credibility of the evidence. Another systematic review and meta-analysis evaluated 60 studies encompassing more than 1.1 million participants. The analysis consistently revealed that greater adherence to the MedDiet was associated with a lower risk of developing T2DM (RR: 0.96; 95% CI: 0.95–0.97), with evidence rated as having moderate to high certainty. The limitations of the study were the heterogeneity of the included studies, risk of bias and confounding, variability in MedDiet definitions, limited long-term data, generalizability constraints, publication bias, and quality of evidence [43].

A cohort study by Ying et al. that included 12,575 participants found that higher adherence to the MedDiet was significantly associated with a lower risk of new-onset diabetes in the Chinese population. Specifically, each incremental increase in the MedDiet adherence score corresponded to a 17% reduction in diabetes risk over a median 9-year follow-up. Additionally, greater consumption of fruits, fish, and nuts was linked to decreased diabetes incidence, and factors such as age, sex, body mass index, and energy intake moderated these associations. The main limitations of this study were the reliance on self-reported physician-diagnosed diabetes and dietary intake data and the unavailability of information on the family history of diabetes [44]. In a study by Sobiecki et al., a nutritional biomarker score based on circulating carotenoids and fatty acids was developed to objectively assess adherence to the MedDiet. Applying this biomarker score in the EPIC-InterAct case–cohort study, which included 22,202 participants with an average follow-up of 9.7 years, higher objectively measured adherence to the MedDiet was strongly associated with a lower risk of T2DM, with a hazard ratio of 0.71 per standard deviation increase in the score after adjusting for multiple confounders, outperforming self-reported dietary adherence (HR 0.90). The findings indicate that a 10-percentile increase in adherence to the MedDiet among Western European adults is associated with an 11% reduction in the incidence of T2DM. This study highlights the importance of using objective biomarkers to better understand the diet–disease relationship. However, the study acknowledged limitations, including potential measurement errors in biomarker assessment, uncertain specificity of the biomarker score to the MedDiet, and the possibility of residual confounding affecting the results [45]. The accumulated body of evidence supports the MedDiet as a highly effective dietary pattern for the prevention of T2DM, demonstrating consistent associations with reduced diabetes incidence across diverse populations and study designs, thereby underscoring its critical role in public health strategies aimed at combating the global diabetes epidemic. Table 1 summarizes the key findings of systematic reviews and meta-analyses that present evidence substantiating the influence of the MedDiet on the prevention of T2DM.

## 4. Effect of the Mediterranean Diet on Glycemic Control in Type 2 Diabetes

This section examines specific studies that elucidate the efficacy of the MedDiet on glycemic control and cardiovascular outcomes in T2DM. A meta-analysis of extended clinical trials demonstrated that following the MedDiet resulted in a notable reduction in HbA1c levels (−0.47%), indicative of enhanced glycemic control in T2DM patients [33]. This finding further substantiates the superiority of the MedDiet over conventional care or low-fat dietary interventions (Figure 2, top panel).

**Figure 3:**
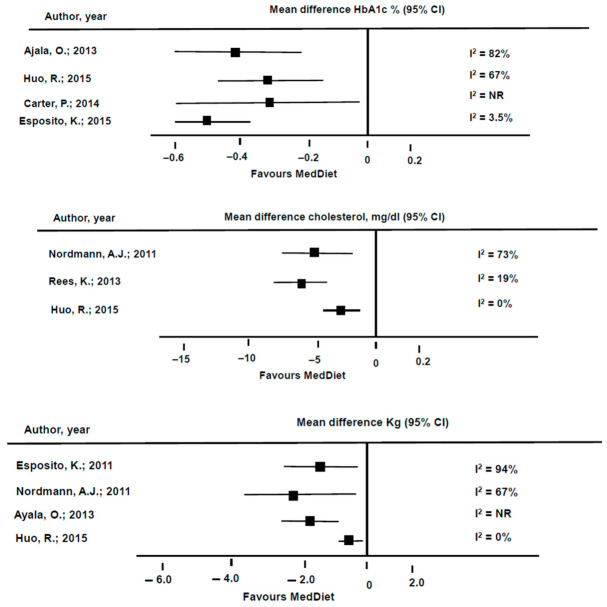
Overview of meta-analyses evaluating the impact of the Mediterranean diet (MedDiet) on HbA1c (top panel) [33,52,53,54], total cholesterol (middle panel) [53,55,56], and body weight (bottom panel) [52,53,55,57].

The top panel of Figure 3 presents a detailed summary of four meta-analyses, one of which is depicted in Figure 2, examining the effects of the MedDiet on glycemic regulation in patients with T2DM. Across all four studies, the MedDiet consistently proved to be more effective than the control diet, as evidenced by the marked reduction in HbA1c levels. Specifically, the reduction in HbA1c levels attributable to the MedDiet ranged from 0.3% to 0.47% [33]. A limitation of this study is the paucity of long-term studies examining MedDiet dietary patterns and their effects on diabetes regulation. Furthermore, this analysis was constrained by the heterogeneous definitions of the MedDiet employed in the reviewed studies. This variability in diet definitions may lead to inconsistencies in the reported outcomes, making it challenging to compare the findings across different studies. The lack of a standardized definition of the MedDiet in research settings can result in varying dietary compositions, potentially affecting the observed effects on diabetes regulation.

A systematic review and meta-analysis conducted by Ajala et al. investigated various dietary strategies for the management of T2DM [52]. The study demonstrated that the MedDiet, low-carbohydrate, low-glycemic index, and high-protein diets have beneficial effects on glycemic control, lipid profiles, and weight reduction [52]. Among these dietary interventions, the MedDiet demonstrated the most substantial improvement in glycemic control. Compared to the control diet, it exhibited notable benefits, including a 0.47% reduction in HbA1c levels, superior weight loss (−1.84 kg), and decreased triglycerides (−0.21 mmol/L). The limitations of this study encompass several key aspects that warrant consideration. The variability in macronutrient composition among the control diets introduced potential confounding factors, thereby complicating the isolation of specific effects attributable to the intervention diet. Furthermore, the heterogeneity in the baseline characteristics of the study participants, particularly regarding weight and HbA1c levels, may have influenced the observed outcomes and potentially limited the generalizability of the findings. The disparities observed may have affected the degree of changes induced by dietary interventions, potentially concealing or intensifying the genuine effects of MedDiet. Moreover, the methodological limitations observed in some of the studies included in the review raise concerns regarding the overall quality and reliability of the evidence. The absence of reported allocation concealment and assessor blinding procedures in certain trials may have introduced bias, potentially compromising the validity of the results. Additionally, the independent effect of weight changes on glycemic control and lipid profiles was a significant confounding factor. This interplay between weight alterations and metabolic parameters impedes the discernment of whether the observed improvements are primarily attributable to dietary intervention itself or to the associated weight loss.

The capacity of the MedDiet to modulate glycemic levels and manage body weight in individuals with T2DM is supported by a comprehensive body of scientific evidence. Studies have highlighted a notable decrease in HbA1c levels, ranging from 0.32% to 0.53%, in individuals who follow the MedDiet as opposed to those on low-fat or control diets, thereby emphasizing its superior effectiveness [44,46,53,54]. The ATTICA study identified a correlation between this dietary plan and improved fasting glucose and insulin levels, as well as a more favorable insulin resistance index in individuals with both normal blood sugar levels and T2DM [58]. In a one-year study, researchers compared the effects of three diets on 259 overweight T2DM patients: a low-carbohydrate MedDiet, a conventional MedDiet, and a diet recommended by the ADA [58]. Individuals who followed these dietary regimens achieved average weight losses of 10.1 kg, 7.4 kg, and 7.7 kg, respectively. Additionally, the MedDiet group exhibited a reduction in HbA1c levels ranging from 0.2% to 0.4%. The findings of these two studies substantiate the positive effects of MedDiet dietary patterns on the aforementioned health parameters.

Table 2 summarizes the key findings of systematic reviews and meta-analyses which present evidence substantiating the influence of the MedDiet on the glycemic control and cardiovascular outcomes in T2DM.

Given the well-documented advantages of the MedDiet in managing glycemic levels, it is imperative to investigate how diet also influences cardiovascular health and cardiovascular risk factors, including arterial hypertension and dyslipidemia, among individuals with T2DM, as examined in the PREDIMED study [35,62]. The PREDIMED study investigated the impact of the MedDiet on the prevention of cardiovascular disease in a population of 7447 individuals, both male and female, aged between 55 and 80 years. All participants were at an elevated risk for cardiovascular events but did not have any cardiovascular disease at baseline. The cohort included individuals diagnosed with T2DM or exhibiting at least three major cardiovascular risk factors, such as smoking, hypertension, dyslipidemia (elevated LDL or reduced HDL cholesterol), excess body weight, or a family history of premature coronary artery disease. Participants were randomly assigned to one of three dietary interventions: a MedDiet enriched with EVOO, a MedDiet supplemented with mixed nuts, or a low-fat diet serving as the control group [35]. The primary endpoint was the incidence of major cardiovascular events, including myocardial infarction, stroke, and cardiovascular mortality. To assess adherence, biomarkers were measured in randomly selected subsets of participants at years one, three, and five. These included urinary hydroxytyrosol levels in the EVOO group and plasma alpha-linolenic acid concentrations in the nut group. The median follow-up duration was 4.8 years (interquartile range, 2.8–5.8 years). The study demonstrated a significant reduction in cardiovascular events among participants adhering to the MedDiet, with incidence rates of 3.8% and 3.4% in the EVOO and nut groups, respectively, compared to 4.4% in the control group. This corresponds to a relative risk reduction of approximately 30%, establishing the MedDiet as an effective intervention for individuals with a high cardiovascular risk. These cardiovascular benefits are likely attributable to the diet’s emphasis on heart-healthy fats and high-fiber foods. A key methodological concern in this study was the modification of the control group’s dietary guidance during the study, which was initially less comprehensive than that provided to the MedDiet groups. This change may have introduced bias favoring the MedDiet arms, although no significant association between enrollment timing and outcomes was identified in this study. Participant attrition, particularly within the control group, posed another challenge; those who withdrew tended to have a less favorable cardiovascular risk profile at baseline, potentially biasing the results. This research underscores the efficacy of the MedDiet as an intervention strategy, emphasizing its role in the prevention of diabetes and cardiovascular disease in individuals at high risk. The findings of this study enhance scientific understanding of how specific dietary patterns can simultaneously address multiple health concerns among high-risk individuals.

Whiteley et al., in an umbrella review, synthesized evidence from 30 systematic reviews, encompassing over 212 randomized controlled trials, to evaluate the comparative effectiveness of various dietary patterns on glycemic control and cardiovascular risk factors in patients with T2DM [63]. Approximately 6875 participants adhering to the MedDiet were included in the four systematic reviews with meta-analyses. This umbrella review established that the MedDiet, compared with a high-carbohydrate low-fat control diet, reduced HbA1c in three of four reviews with a mean difference (MD) between 0.3% and 0.5%, and in one review, the reduction in HbA1c was also seen, with an MD of −0.1%, but it was statistically insignificant. Regarding fasting plasma glucose levels, two of the systematic reviews with meta-analyses demonstrated that adherence to the MedDiet resulted in significantly greater reductions compared to a low-fat control diet, with mean differences ranging from 0.6 to 0.7 mmol/L.

Weight changes associated with the MedDiet were examined in four systematic reviews that incorporated meta-analyses. Two of these reviews demonstrated that the MedDiet resulted in a significantly greater reduction in weight compared to the control diet, with average decreases ranging from 1.6 to 1.8 kg. In contrast, the other two reviews reported no significant differences between diets. Furthermore, two systematic reviews with meta-analyses evaluated cardiovascular risk factors, showing significant improvements in blood pressure, total cholesterol, triglycerides, and HDL cholesterol when the MedDiet was compared with relevant control diets. This study had several limitations, including insufficient reporting on adherence to dietary patterns and duplication of data from individual randomized controlled trials. The findings indicate that adherence to the MedDiet is associated with modest but consistent improvements in glycemic control among patients with T2DM.

A meta-analysis conducted by Zheng et al., encompassing seven RCTs with a total of 1371 participants, demonstrated that adherence to the MedDiet significantly enhanced glycemic control in individuals with T2DM [64]. This was evidenced by a reduction in HbA1c levels (MD = −0.39%, 95% CI: −0.58 to −0.20) and fasting plasma glucose (MD = −0.84 mmol/L, 95% CI: −24.69 to −5.55), indicating clinically meaningful improvements compared with the control diet. Although the MedDiet did not produce statistically significant alterations in total cholesterol, HDL, or LDL cholesterol levels compared to the control diets, it was associated with a reduction in both diastolic and systolic blood pressure levels. This study was limited by the composition of the control group, which included individuals with diverse dietary habits, potentially influencing the results. Additionally, the limited number of trials restricted the ability to perform further subgroup analyses of specific variables.

In a network meta-analysis that included ten RCTs, Pan et al. comprehensively compared the efficacy of five dietary patterns on key clinical outcomes in patients with T2DM [65]. The Mediterranean diet showed significant benefits over the low-fat diet in improving glycemic control. Evidence showed a reduction in HbA1c levels (MD = −0.45%, 95% CI: −0.55 to −0.34) and a decrease in fasting plasma glucose (MD = −1.24 mmol/L, 95% CI = −1.57 to −0.91). The MedDiet, compared with a low-fat diet, was also associated with significant reductions in body weight, waist circumference, total cholesterol, HDL cholesterol, and triglyceride levels. This network meta-analysis supported the MedDiet as the most effective dietary pattern among those examined for improving glycemic control, achieving modest weight loss, and improving cardiovascular risk factors in patients with T2DM. However, owing to the limited number of trials, variability in study designs, and insufficient data on long-term outcomes, these findings should be interpreted with caution.

The meta-analysis of 11 RCTs by Wu et al. demonstrated that the MedDiet significantly improved glycemic control in patients with T2DM, as evidenced by reductions in HbA1c levels (MD = −0.307%, 95% CI: −0.451 to −0.163) and fasting plasma glucose levels (MD = −0.845 mmol/L, 95% CI: −1.307 to −0.384) compared with control diets [66]. The MedDiet also positively influenced body mass index, LDL cholesterol, as well as systolic and diastolic blood pressure. The limitations of this meta-analysis include the predominance of trials conducted in Mediterranean or Western regions, resulting in limited representation of participants from non-Mediterranean dietary cultures. This geographical concentration may constrain the generalizability of the findings to the global population. Furthermore, variations in intervention intensity may have influenced the outcomes. Additionally, there was considerable variability in the duration of follow-up among the trials, several trials had small sample sizes, and the definitions of the MedDiet varied across the studies.

A systematic review and meta-analysis of intervention trials by Lauria et al. included nine RCTs with a total of 1337 participants. In comparison to control diets, interventions involving the MedDiet resulted in a significant reduction in HbA1c levels, with an MD of −0.18% (95% CI = −0.35, −0.01), as well as decreases in LDL cholesterol by −0.10 mmol/L (95% CI = −0.19, −0.00) and triglycerides by −0.20 mmol/L (95% CI = −0.28, −0.12). However, no significant alterations were observed in fasting plasma glucose, total cholesterol, or HDL cholesterol levels [67]. The findings suggest that interventions incorporating the MedDiet may enhance HbA1c, LDL cholesterol, and triglyceride levels, thereby potentially contributing to the diet’s metabolic benefits. This study had several limitations, including the small sample sizes and short durations of many of the original studies. Furthermore, there was considerable heterogeneity due to variations in dietary interventions, initial health conditions, sample sizes, and differences in the lengths of interventions, which may have affected the generalizability of the findings.

Evidence consistently demonstrates that adherence to the MedDiet results in significant improvements in glycemic control and cardiovascular outcomes among individuals with T2DM. Numerous meta-analyses and systematic reviews have documented reductions in HbA1c levels ranging from approximately 0.3% to 0.5%, along with improvements in fasting plasma glucose. Additionally, the MedDiet has been shown to positively influence lipid profiles and contribute to weight loss, which further supports metabolic health. The PREDIMED study further corroborated the role of the MedDiet in reducing major cardiovascular events by approximately 30% in high-risk populations, underscoring its multifaceted benefits beyond glycemic regulation [35]. Nonetheless, limitations such as heterogeneity in MedDiet definitions, variability in control diets, participant characteristics, study designs, and geographic and demographic constraints temper the generalizability and long-term applicability of these findings. Future research should aim to standardize MedDiet parameters, incorporate the use of nutritional biomarker scores for the objective assessment of adherence, and expand investigations across diverse populations and longer durations to solidify the role of the MedDiet as a cornerstone dietary intervention in T2DM management and cardiovascular risk reduction.

## 5. Introduction to Mechanisms: How the Mediterranean Diet Affects Type 2 Diabetes

This section explores the pathways by which the MedDiet aids T2DM management and prevention. Prior to examining dietary interventions, a thorough understanding of T2DM pathophysiology is imperative to understand how certain dietary approaches, particularly the MedDiet, counteract the underlying pathophysiological mechanisms of the disease. T2DM is characterized by chronic hyperglycemia, which stems from either inadequate secretion of insulin by pancreatic β-cells, the presence of insulin resistance in cells, or a combination of both. This condition arises when insulin is not produced in sufficient amounts or fails to effectively promote glucose uptake into cells. When the body improperly manages carbohydrates, fats, and proteins, it can result in severe blood vessel complications, both microvascular and macrovascular [68]. In addition to genetic abnormalities and aging, various other factors may contribute to the impairment of insulin secretion by β-cells. These factors include glucotoxicity, lipotoxicity, oxidative stress, activation of inflammatory pathways, and insulin resistance, all of which can result in β-cell stress and/or a diminished incretin effect on β-cells [69,70,71,72]. Upon establishing insulin resistance as a critical component in T2DM, our investigation explored the MedDiet’s potential to effectively ameliorate this resistance. Within the spectrum of dietary interventions, the MedDiet has gained recognition owing to its potential efficacy. Its unique components are capable of influencing pathways that are integral to mitigating insulin resistance and the dysfunction of β-cells. Specifically, unsaturated free fatty acids found in the MedDiet directly enhance insulin sensitivity by facilitating glucose uptake in cells, whereas polyphenols reduce oxidative stress, thereby addressing two key factors in T2DM pathophysiology and reducing the risk of metabolic syndrome, T2DM, and cardiovascular diseases [24].

### 5.1. Anti-Inflammatory and Antioxidant Pathways: Underlying Mechanisms

The MedDiet pattern, renowned for its health-enhancing qualities, is characterized by an abundance of nutrient-rich foods that diminish inflammatory processes and counteract oxidative stress. Adherence to the MedDiet pattern elevates plasma antioxidant levels [73], reduces C-reactive protein (CRP) levels [74], and mitigates the impact of acute hyperglycemia on endothelial function, oxidative stress, and inflammation [75]. Central to the anti-inflammatory and antioxidant properties of the MedDiet are its bioactive compounds, among which phenolic compounds (PCs), particularly flavonoids such as quercetin, play a crucial role. Quercetin, a key flavonoid in this diet, activates the adenosine monophosphate-activated protein kinase (AMPK) pathway in muscle cells [76]. This pathway is crucial for increasing glucose uptake, a process that remains effective even when the body experiences oxidative stress [77]. In addition to flavonoids, the abundance of polyphenols in the diet, particularly those present in olive oil, further emphasizes its health benefits. Polyphenolic compounds are found not only in olive oil but also in legumes, vegetables, fruits, and cereals. Olive oil is rich in oleuropein, hydroxytyrosol, and tyrosol, particularly EVOO. Oleuropein, hydroxytyrosol, and tyrosol are essential in mitigating oxidative stress by blocking pro-inflammatory mediators and enhancing nitric oxide availability, which improves endothelial function [78]. Among the sources of PCs and flavonoids, EVOO is notable for its high concentration as well as its distinctive molecules that further enhance these anti-inflammatory and antioxidant effects. EVOO is abundant in polyunsaturated fatty acids (PUFAs), such as linoleic and α-linolenic acids, which improve adipose tissue inflammatory responses and insulin sensitivity [79]. In the context of obesity, adipose tissue exhibits dysfunctionality, leading to the production of various cytokines and chemokines that facilitate inflammation. Notable among these are tumor necrosis factor alpha, interleukin-6, and resistin, which activate intracellular signaling pathways that contribute to insulin resistance in tissues targeted by insulin [75].

Monounsaturated fatty acids (MUFAs), predominantly found in EVOO (especially oleic acid), counteract the effects of saturated fatty acids (SFAs) that decrease peripheral tissue insulin sensitivity [80,81]. By mitigating the effects of deleterious lipids, MUFAs not only enhance biochemical processes but also contribute to measurable health outcomes, such as improved insulin sensitivity and a potential reduction in the risk of T2DM [82]. The MedDiet, which includes flavonoids found in PCs and MUFAs present in olive oil, is well regarded for its anti-inflammatory and antioxidant effects. These dietary components are instrumental in lowering inflammatory markers and improving insulin sensitivity. While the fatty acids in EVOO are essential in modulating inflammation, the antioxidant components of the oil, such as α-tocopherol and carotenoids, add another layer of protective effects [83]. EVOO is abundant in α-tocopherol, carotenoids, phytosterols [83], and omega-3 fatty acids, and its increased consumption is associated with a reduction in triglyceride levels and circulating inflammatory markers [75]. Individuals with insulin resistance exhibit increased susceptibility to developing T2DM, and interventions focusing on fundamental lifestyle modifications, including the adoption of the MedDiet, can contribute to the mitigation of this risk.

### 5.2. Contrasting Effects: The Role of Branched-Chain Amino Acids in Metabolic Health

Metabolic health is essential for the prevention of diseases such as T2DM and obesity. While PUFAs contribute positively to metabolic health, BCAAs and their derivatives have been linked to negative health outcomes, including insulin resistance, T2DM, cancer, and cardiovascular diseases [84]. Understanding the role of BCAAs begins by recognizing their elevated presence within an organism and their subsequent detrimental effects on insulin sensitivity. Elevated levels of BCAAs, specifically valine, leucine, and isoleucine, may contribute to insulin resistance, possibly through activation of a cellular pathway known as mammalian target of rapamycin (mTOR) complex 1 [85]. They also stimulate the transcription factor nuclear factor kappa B (NF-κB), resulting in the secretion of pro-inflammatory molecules, such as intercellular adhesion molecule 1 (ICAM-1), tumor necrosis factor α (TNF-α), and interleukin 6 (IL-6) [86]. Research indicates that regular consumption of EVOO, through its PUFA content, may mitigate the adverse effects of BCAAs by reducing their plasma levels [87], potentially through mechanisms that warrant further investigation of their implications for metabolic health. Through the reduction in BCAA levels, EVOO consumption directly complements the insulin-regulating effects of GLP-1 and the anti-inflammatory properties of PUFAs, thereby providing a multifaceted approach for improving metabolic health. A comprehensive understanding of dietary influences on metabolic health elucidates the complex interplay between beneficial components, such as GLP-1 and PUFAs, and potentially deleterious components, such as BCAAs. Future research should aim to elucidate these complex mechanisms and develop targeted dietary interventions for the management of T2DM and obesity.

### 5.3. Modulation of Intestinal Microbiota as a Potential Pathway

The composition of the gastrointestinal microbiota changes throughout an individual’s lifespan, and significant variations have been observed among individuals and across different geographical regions. The critical significance of the gastrointestinal microbiome in regulating human physiological homeostasis provides a conceptual framework for investigating how specific nutritional interventions, such as the MedDiet patterns, can effectively address microbial dysbiosis. Disruption of the intestinal microbiota balance can adversely affect human homeostasis, as evidenced by observational studies demonstrating its potential role in various conditions, including T2DM [88,89,90]. In individuals with T2DM, cross-sectional and associative data reveal that the composition of the gut microbiota undergoes significant alterations, including a reduction in butyrate-producing bacteria (Firmicutes and others) [91,92,93] and an increase in sulfate-reducing bacteria [92,93]. Specifically, a metagenome-wide association study identified a moderate state of gut microbial imbalance as a key feature of T2DM. This condition manifests through a reduction in common butyrate-producing strains, an influx of opportunistic pathogens, and an enrichment of specific microbial functions that support both sulphate reduction and resistance to oxidative stress [94]. Observational and cohort data suggest that adherence to the MedDiet is associated with an enhanced abundance of beneficial bacteria, such as Firmicutes, which produce butyrate, a compound crucial for intestinal health, and influences the equilibrium of the key gut phyla Firmicutes and Bacteroidetes [94]. Therefore, this dietary approach may provide a strategic method for preventing and treating chronic diseases, hypothetically by modulating gut microbiota composition [95]. Furthermore, metabolomic profiling and associative evidence indicate that the MedDiet may shift the composition of the gut microbiota, which is linked to a reduction in the abundance of certain microorganisms, such as Prevotella copri and Bacteroides vulgatus. In preclinical and associative models, these strains have been associated with elevated BCAA levels and insulin resistance [75,96]. Consequently, this dietary pattern is suggested to exert a specific influence on metabolic pathways. Understanding the effect of diet on gut microbiota composition establishes the foundation for examining its significant role in the production of short-chain fatty acids (SCFAs), which are crucial in the management of T2DM. Owing to its high fiber content, the MedDiet is associated with enhanced gut fermentation processes, thereby increasing the production of beneficial SCFAs, including acetate, butyrate, and propionate [75]. Mechanistic and experimental data indicate that these SCFAs are integral to the effective management of T2DM as they exert a significant influence on the regulation of lipid and glucose metabolism, potentially through the activation of specific receptors situated at key metabolic locations, including the liver, pancreas, adipose tissue, and brain [97]. Furthermore, experimental models demonstrate that SCFAs facilitate the secretion of GLP-1 and GLP-2, which plausibly results in improved insulin sensitivity, enhanced proliferation of pancreatic β-cells, and increased satiety. In line with this, data from both experimental models and targeted human interventions indicate that alterations of the gut microbiome using pre- and probiotics can play a key role in managing metabolic conditions linked to obesity [98,99]. Notably, individuals with T2DM demonstrate SCFA deficiencies in clinical observations [100]. Consequently, the MedDiet presents a biologically plausible benefit for this patient population because its high dietary fiber content has been shown in dietary intervention trials to stimulate SCFA production by the gut microbiota [75].

### 5.4. Mechanism Related to Carbohydrates, Fiber and Minerals

Plant-based foods play a crucial role in promoting health through various mechanisms, primarily through their carbohydrate, fiber, and mineral contents. Plant-based foods, particularly seeds (cereals, legumes, and nuts), are essential sources of slow-release carbohydrates and dietary fiber. Dietary fiber can be classified into two categories. The first category comprises metabolically inert insoluble fibers (e.g., cellulose and lignin), which are predominantly derived from cereal grains. The second category consists of bioactive soluble fibers (e.g., pectin and gums) found in fruits and vegetables. According to multiple studies, a higher intake of dietary fiber is directly correlated with a reduced risk of T2DM, increased satiety, reduced blood pressure, and improved cardiovascular health [19,101,102,103]. In addition to dietary fiber, the presence of essential minerals in plant-based foods significantly enhances their health benefits. These minerals coexist with fibers and substantially augment the overall health benefits of fiber consumption, including improved insulin sensitivity and cardiovascular health [102]. Plant seeds, such as cereals, legumes, and nuts, are notably low in sodium and rich in potassium (K^+^), magnesium (Mg^2+^), and calcium (Ca^2+^). Each of these minerals present in plant seeds performs a distinct function in the human body, ranging from osseous tissue formation to the regulation of neural and muscular activity and fluid homeostasis [104]. One significant benefit of increasing magnesium intake is the enhancement of insulin sensitivity, which reduces the risk of T2DM [105].

### 5.5. Sterol-Based Mechanism

Although plant-based foods do not contain cholesterol, the lipid components of nuts and oils are abundant in phytosterols, which are structurally similar to cholesterol and perform essential functions in cellular membranes [106]. Phytosterols, even in small quantities, can inhibit the absorption of cholesterol in the intestinal tract, resulting in a reduction in overall cholesterol levels [107,108]. In the context of T2DM management, the capacity of phytosterols to modestly lower triglyceride levels [108] is significant, as elevated triglyceride levels are a well-documented risk factor for cardiovascular disease among these individuals. Consequently, the abundance of these beneficial compounds in the MedDiet effectively improves blood lipid profiles and positively influences health outcomes in T2DM [106].

### 5.6. Alcohol-Based Mechanism

Moderate alcohol consumption, particularly red wine, is another promising component in T2DM management due to its unique cardiovascular benefits. These benefits include the enhancement of cardiovascular health, a critical aspect of T2DM management, and the potential modulation of postprandial blood glucose levels [109]. Among alcoholic beverages, red wine is notable for its exceptional capacity to enhance HDL cholesterol levels. HDL, often termed ‘good’ cholesterol, is integral to the maintenance of cardiovascular health. The high polyphenol content in red wine plays a key role in its effectiveness in T2DM management by improving cardiovascular health [110]. This beverage exerts a dual therapeutic effect: it facilitates blood pressure reduction by enhancing nitric oxide availability while concurrently attenuating postprandial glycemic excursions by mitigating oxidative stress [110]. These physiological processes highlight the potential benefits of incorporating red wine consumption with meals as an adjunctive intervention into T2DM management strategies, contributing to improved blood pressure regulation and glycemic control. Although moderate red wine consumption offers significant benefits for T2DM management, it is imperative to approach its inclusion in the diet with caution to mitigate the risks associated with excessive alcohol intake, such as liver disease, addiction, and increased susceptibility to other chronic conditions. A balanced approach to T2DM management requires careful consideration of these factors.

## 6. The Mediterranean Diet in the Era of Glucagon-like Peptide-1 Agonists and Sodium–Glucose Cotransporter-2 Inhibitors

As previously mentioned, the MedDiet increases GLP-1 levels, which is significant due to the hormone’s multifaceted role in metabolic health. Compared to a high-fiber vegetarian diet, GLP-1 and oxyntomodulin levels are significantly higher following the MedDiet [111]. Oxyntomodulin is a peptide hormone secreted together with GLP-1 from the gut after food ingestion; it stimulates glucagon and GLP-1 receptors, leading to a reduction in body weight. Oxyntomodulin also reduces food intake and increases energy expenditure [112]. The MedDiet may significantly enhance the effects of GLP-1 receptor agonist therapy by improving long-term adherence, reducing metabolic adaptation, and supporting sustained weight loss. While GLP-1 agonists effectively suppress appetite and reduce body weight, they do not substantially increase energy expenditure or prevent the decline in basal metabolic rate that often accompanies weight reduction. The MedDiet, particularly when combined with selected low-carbohydrate or ketogenic elements, may further improve insulin sensitivity, glycemic control, and inflammatory markers while helping preserve lean muscle mass during weight loss. In addition, this dietary pattern appears to be more sustainable in the long term and may help maintain metabolic benefits even after discontinuation of GLP-1 therapy [113]. The MedDiet, particularly through its high content of polyunsaturated fatty acids (PUFAs) derived from extra-virgin olive oil, may enhance endogenous GLP-1 secretion by directly activating intestinal G-protein-coupled receptors such as GPR120. In addition, the antioxidant and anti-inflammatory properties of the MedDiet components reduce oxidative stress and chronic low-grade inflammation, mechanisms implicated in the development of GLP-1 resistance. These effects may improve incretin responsiveness and contribute to better glycemic control and metabolic outcomes in patients receiving GLP-1 receptor agonist therapy [75]. The MedDiet may represent an ideal nutritional foundation for patients treated with GLP-1 receptor agonists such as semaglutide. Owing to its anti-inflammatory properties, high fiber content, and emphasis on minimally processed foods, this dietary pattern may help alleviate common gastrointestinal adverse effects of GLP-1 therapy, including nausea and constipation. The synergistic interaction between semaglutide therapy and the MedDiet promotes gradual, sustainable, and metabolically healthy weight loss while reducing the risk of malnutrition and excessive lean body mass depletion [114]. Finally, recent evidence suggests that adherence to the MedDiet may potentiate the metabolic benefits of dual GIP/GLP-1 receptor agonist therapy with tirzepatide. Patients with high adherence to a MedDiet pattern achieved significantly greater reductions in the visceral adiposity index compared with those relying primarily on pharmacological treatment alone. These findings indicate that the MedDiet may synergistically enhance the effects of tirzepatide on visceral fat reduction, likely through its anti-inflammatory properties, improved insulin sensitivity, and favorable effects on lipid metabolism. Such combined lifestyle–pharmacological approaches may therefore provide superior cardiometabolic benefits in patients with obesity and T2DM [115].

The MedDiet and SGLT-2 inhibitors appear to exert complementary metabolic effects that may synergistically improve glycemic regulation and cardiometabolic health in T2DM. While SGLT-2 inhibitors reduce plasma glucose levels through enhanced urinary glucose excretion, the MedDiet improves insulin sensitivity, attenuates postprandial glycemic excursions, and reduces chronic low-grade inflammation and oxidative stress. Together, these interventions may provide a more stable metabolic environment and contribute to improved long-term glycemic control. In addition to their glucose-lowering properties, both the MedDiet and SGLT-2 inhibitors exert significant nephroprotective effects. Their combined use may therefore maximize nephron protection and slow the progression of diabetic kidney disease [116]. The MedDiet may synergistically enhance the therapeutic effects of SGLT-2 inhibitors on reductions in systolic blood pressure. Patients combining SGLT-2 inhibitors with the MedDiet achieved an additional reduction in systolic blood pressure of approximately 5 mmHg, indicating clinically relevant cardiovascular benefits [66]. Patients combining SGLT-2 inhibitors with the MedDiet may achieve an additional weight loss of approximately 3–5 kg compared with either pharmacological therapy or dietary intervention alone. These benefits are likely mediated through complementary mechanisms, including increased urinary glucose excretion, improved insulin sensitivity, appetite regulation, and reduced caloric intake associated with the MedDiet [117]. Most importantly, in contrast to restrictive low-carbohydrate or ketogenic diets that increase the risk of euglycemic diabetic ketoacidosis, adherence to the MedDiet may provide a more metabolically stable and protective nutritional environment and does not appear to increase the risk of diabetic ketoacidosis in patients treated with SGLT-2 inhibitors. Owing to its balanced macronutrient composition and moderate carbohydrate intake, the MedDiet dietary pattern is considered one of the safest nutritional approaches in patients receiving SGLT-2 inhibitor therapy.

## 7. The Mediterranean Diet in Non-Mediterranean Populations

The implementation of the MedDiet in non-Mediterranean populations requires cultural adaptation while preserving its core nutritional principles. Rather than focusing exclusively on traditional Mediterranean foods, dietary recommendations may emphasize locally available, culturally acceptable, and affordable foods with similar nutritional profiles [118]. For example, when access to extra-virgin olive oil, nuts, fish, or fresh produce is limited, alternative sources of unsaturated fats, plant-based proteins, whole grains, and seasonal fruits and vegetables may be incorporated. High-quality rapeseed oil may substitute for olive oil, while canned or frozen fish can provide comparable amounts of omega-3 fatty acids. Similarly, frozen vegetables, seasonal local produce, legumes, and seeds may serve as cost-effective alternatives to more expensive fresh produce, nuts, and traditional Mediterranean foods, thereby improving accessibility across different socioeconomic settings [119]. This approach may improve adherence while reducing economic barriers and increasing long-term sustainability. Importantly, socioeconomic status significantly influences dietary choices, as healthier food options are often perceived as more expensive or less accessible. Recent evidence suggests that adherence may be improved by emphasizing the fundamental principles of the MedDiet rather than the strict consumption of traditional Mediterranean foods [43]. Therefore, adapting the MedDiet pattern to local food environments and cultural preferences may help reduce health disparities and facilitate broader implementation across diverse populations. Furthermore, community-based interventions, educational programs, and practical dietary guidance may support the adoption and maintenance of Mediterranean-style eating habits outside Mediterranean regions [120].

## 8. Limitations

This review has several limitations that should be acknowledged. First, the definition of the MedDiet varied across the included studies, with differences in dietary components, scoring systems, and adherence criteria, which may have contributed to heterogeneity in the reported outcomes. Second, many studies relied on self-reported dietary intake data, making the results susceptible to recall bias, reporting inaccuracies, and misclassification of dietary adherence. Third, a substantial proportion of the available evidence originated from observational studies, which are inherently vulnerable to residual confounding despite statistical adjustment for known risk factors. Furthermore, the use of different MedDiet adherence scores across studies limited direct comparisons and may have influenced the magnitude of observed associations. Finally, most studies were conducted in Mediterranean or Western populations, potentially limiting the generalizability of the findings to other geographic regions, ethnic groups, and cultural settings with different dietary patterns and lifestyles. Future research should prioritize standardized definitions of the MedDiet, objective measures of dietary adherence, and well-designed randomized controlled trials in diverse populations to strengthen the evidence base and improve the applicability of findings worldwide.

## 9. Conclusions

The MedDiet exerts a substantial influence on the prevention and management of T2DM, as evidenced by systematic reviews and meta-analyses, through multiple mechanisms, including anti-inflammatory and antioxidant effects, the presence of endogenous GLP-1 receptor agonist-stimulating compounds, and the modulation of the gut microbiome. This dietary approach serves as an effective tool for the prevention of T2DM, optimization of glycemic control, particularly in combination with modern medications, and management of complications. The effectiveness of the MedDiet stems from the complex interplay between its diverse nutrient components and their metabolic byproducts. Moreover, this nutritional strategy plays a pivotal role in addressing metabolic syndrome and in attenuating the risks associated with obesity, cardiovascular disorders, malignancies, and cognitive decline. The implementation of a nutritionally comprehensive diet in conjunction with consistent physical activity is essential for promoting holistic health and enhancing overall quality of life. Future research recommendations encompass the necessity for long-term randomized controlled trials to critically evaluate the applicability of the established health benefits of the MedDiet across diverse populations with varying dietary habits and cultural backgrounds.

## Figures and Tables

**Table 1:** Relationship between the Mediterranean diet and prevention of type 2 diabetes.

First Author; Year; Reference	Type of Study	Type of Intervention; Participants	Output(s); Follow Up	Results
Esposito, K.; 2014 [36]	MA 8 prospective studies, 30 cohorts	MedDiet, DASH Adults > 20 years *n* = 21 372	Incidence of T2DM 3.2–23 years	The RR associated with a healthy diet was 0.80, with a 95% CI ranging from 0.74 to 0.86. There were no statistically significant differences in the incidence rates of T2DM when comparing the MedDiet with the DASH diet.
Koloverou, E.; 2014 [32]	SR, MA 1 RCT, 9 prospective studies	MedDiet, Control diet Healthy adults with or without CV/T2DM *n* = 136,846	Incidence of T2DM 3.5–14 years	A higher level of adherence to the MedDiet is linked to a 23% decrease in the risk of developing T2DM. This association is supported by a combined relative risk of 0.77 (95% CI: 0.66, 0.89) when comparing individuals in the highest adherence centile with those in the lowest.
Schwingshackl, L.; 2015 [46]	SR, MA 1 RCT 8 prospective studies	MedDiet Healthy adults or with CV risk factors *n* = 122,810	Incidence of T2DM 3.2–20 years	Adherence to MedDiet was inversely correlated with a reduction in the incidence of T2DM, as evidenced by an RR of 0.81 (95% CI: 0.73, 0.90; *p* < 0.0001) when comparing high adherence to low adherence.
Jannasch, F.; 2017 [47]	SR, MA 48 prospective studies: 16 cohorts/ACRs	MedDiet, DASH, AHEI Healthy adults *n* ~ 1.5 millions	Incidence of T2DM 4.1–23 years	MedDiet (RR quantiles: 0.87; 95% CI: 0.82, 0.93), the DASH diet (RR: 0.81; 95% CI: 0.72, 0.92), and the AHEI (RR: 0.79; 95% CI: 0.69, 0.90) were linked with a notable decrease in the risk of T2DM.
Zheng, M.; 2018 [48]	SR, 6 RCTs	Modified MedDiet Adults with obesity	Glycemic control HbA1c >3 months	A low-carbohydrate MedDiet and a MedDiet incorporating virgin olive oil demonstrated a beneficial effect on the prevention of T2DM
Sarsangi, P.; 2022 [49]	SR, MA 16 prospective studies	MedDiet Adults, general population *n* = 759,806	The incidence of T2DM, diet adherence 3.5–25 y	Individuals exhibiting the highest levels of adherence to the MedDiet demonstrated a significantly reduced risk of developing T2DM compared to those with the lowest adherence (pooled RR: 0.83; 95% CI: 0.77, 0.90; I2 = 79%, *p* ≤ 0.001). A linear dose–response analysis indicated that each 1-point increase in the MedDiet score was associated with a 3% reduction in T2DM risk (RR = 0.97; 95% CI: 0.96, 0.98; *p* ≤ 0.001). Additionally, an inverse nonlinear relationship was identified between the MedDiet score and T2DM risk, with the inverse association becoming more pronounced at higher MedDiet scores.
Zeraattalab-Motlagh S; 2022 [50]	SR, MA 14 prospective cohort studies	MedDiet Adults, general population *n* = 410,303	The incidence of T2DM, diet adherence 3–23 y	An inverse association was observed when comparing the highest and lowest adherence levels to the MedDiet, with an RR of 0.79 (95% CI: 0.72, 0.88; I2 = 82%, n = 14; risk difference: −21 per 1000 individuals, 95% CI: −28, −12; GRADE = moderate certainty). In addition, an increase of 2 points in the MedDiet adherence score corresponded to an RR of 0.86 (95% CI: 0.82, 0.91; n = 13)
Martinez-Lacoba, R.; 2018 [51]	SMR 9 SRs, 24 MAs	MedDiet Adults	Diet adherence, obesity, body weight T2DM >6 months	In conclusion, adherence to an MedDiet pattern may contribute to the prevention and management of T2DM

MedDiet = Mediterranean diet, HPD = high-protein diet, BMI = body mass index, AHEI = alternate healthy eating index, DASH = dietary approach to stop hypertension, LGI = low-glycemic index, LC = low carb, CI = confidence interval, CVD = cardiovascular disease, CHO = carbohydrate, HbA1c = glycated hemoglobin A1c, RR = relative risk, CHD = coronary heart disease, MI = Myocardial infarction, SR = structured review, MA = meta-analysis, RCT = randomized controlled trial, SMR = systematic meta-review, MD = mean difference, T2DM = type 2 diabetes mellitus.

**Table 2:** Effect of the Mediterranean diet on glycemic control and cardiovascular outcome in type 2 diabetes.

First Author; Year; Reference	Type of Study	Type of Intervention; Participants	Output(s); Follow Up	Results
Ajala, O.; 2013 [52]	20 RCTs	LC, LGI, MedDiet, HPD Adults with T2DM or obesity	HbA1c, Weight loss >6 months	All dietary interventions led to improvements in glycemic indices, with reductions in HbA1c recorded as follows: −0.12% for the LC diet (*p* = 0.04), −0.14% for the Low GI diet (*p* = 0.008), −0.47% for the MedDiet (*p* < 0.00001), and −0.28% for the HPD (*p* < 0.00001). Both the LC and MedDiets were associated with significant weight loss, amounting to −0.69 kg (*p* = 0.21) and −1.84 kg (*p* < 0.00001), respectively. An increase in high-density lipoprotein levels was observed across all dietary plans, with the exception of the high-protein diet.
Carter, P.; 2014 [54]	SR, MA 8 RCTs	MedDiet, Paleo diet, Control diet Overweight and/or high cardiovascular risk and/or T2DM *n* = 2789	Glycemic control, HbA1c, insulin 2–12 months	MedDiet resulted in a reduction in HbA1c levels when compared to the control group; however, it did not demonstrate superiority over the paleo-diet. Furthermore, none of the dietary interventions showed a significant advantage over the other interventions in terms of basal glucose levels
Huo, R.; 2015 [53]	MA 9 RCTs	MedDiet Adults with T2DM *n* = 1178	Glycemic control. HbA1c, insulin, HOMA-IR 1 month–4 years	Compared to the control group, the MedDiet intervention led to a decrease in HbA1c (median difference −0.30; 95% CI −0.46, −0.14), glucose levels (−0.72 mmol/L; CI −1.24, −0.21), and baseline insulin (−0.55 μU/mL; CI −0.81, −0.29).
Esposito, K.; 2015 [33]	SR 8 MA, 5 RCTs	MedDiet, Control diet Adults with T2DM or at risk *n* = 2087	Incidence of T2DM Glycemic control >6 months	MedDiet enhances glycemic control by reducing HbA1c levels by 0.3–0.47% in comparison to a low-fat diet. Additionally, adherence to the MedDiet correlates with a significant decrease in the incidence of future T2DM, with reductions ranging from 19% to 23%.
Emadian, A.; 2015 [59]	SR 11 RCTs	MedDiet, Vegan diet, LGI diet Overweight adults (BMI ≥ 25 kg/m^2^) and T2DM	Glycemic control, HbA1c >6 months	All dietary groups demonstrated beneficial effects, as evidenced by an improvement in overall glycemic control through a reduction in HbA1c levels
Schwingshackl, K.; 2018 [60]	SR, MA 56 RCTs	Low-fat diet or vegan diet, MedDiet, LC, paleolithic hyperprotein diet Adults with T2DM *n* = 4937	Glycemic control HbA1c 3–48 months	In comparison to the low-fat diet, there was a reduction in HbA1c levels in the MedDiet group (−0.32, 95% CI: −0.53, −0.11) and the LC group (−0.35, 95% CI: −0.56, −0.14). Additionally, there was a decrease in glycemia in the MedDiet group (−0.59 mmol/L, 95% CI: −1.13, −0.04)
Becerra-Tomas, N.; 2019 [61]	SR, MA 3 RCTs, 38 cohorts	MedDiet Adults with T2DM	CVD incidence, myocardial infarction, CVD mortality, coronary heart disease incidence >6 months	MedDiet is associated with a reduced risk of developing total CVD, as indicated by an RR of 0.62 (95% CI: 0.50, 0.78). Similarly, adherence to this diet correlates with a decreased incidence of MI, with an RR of 0.65 (95% CI: 0.49, 0.88). A comparative analysis of individuals with the highest versus lowest adherence to the MedDiet reveals a significant inverse association with total CVD mortality (RR: 0.79; 95% CI: 0.77, 0.82), incidence of CHD (RR: 0.73; 95% CI: 0.62, 0.86), CHD mortality (RR: 0.83; 95% CI: 0.75, 0.92), stroke incidence (RR: 0.80; 95% CI: 0.71, 0.90), stroke mortality (RR: 0.87; 95% CI: 0.80, 0.96), and MI incidence (RR: 0.73; 95% CI: 0.61, 0.88).

MedDiet = Mediterranean diet, HPD = high-protein diet, BMI = body mass index, AHEI = alternate healthy eating index, DASH = dietary approach to stop hypertension, LGI = low-glycemic index, LC = low carb, CI = confidence interval, HOMA = CVD = cardiovascular disease, CHO = carbohydrate, HbA1c = glycated hemoglobin A1c, RR = relative risk, CHD = coronary heart disease, MI = Myocardial infarction, SR = structured review, MA = meta-analysis, RCT = randomized controlled trial, SMR = systematic meta-review, MD = mean difference, HOMA-IR = Homeostatic Model Assessment of Insulin Resistance, T2DM = type 2 diabetes mellitus.

## Data Availability

No new data were created or analyzed in this study. Data sharing is not applicable to this article.

## References

[1] Tosti V., Bertozzi B., Fontana L. (2018). Health Benefits of the Mediterranean Diet: Metabolic and Molecular Mechanisms. J. Gerontol. A Biol. Sci. Med. Sci..

[2] Fundación Dieta Mediterránea. https://dietamediterranea.com/.

[3] Guasch-Ferré M., Willett W.C. (2021). The Mediterranean diet and health: A comprehensive overview. J. Intern. Med..

[4] Keys A. (1980). Seven Countries: A Multivariate Analysis of Death and Coronary Heart Disease.

[5] Schellenberg E.S., Dryden D.M., Vandermeer B., Ha C., Korownyk C. (2013). Lifestyle interventions for patients with and at risk for type 2 diabetes: A systematic review and meta-analysis. Ann. Intern. Med..

[6] Viigimaa M., Sachinidis A., Toumpourleka M., Koutsampasopoulos K., Alliksoo S., Titma T. (2020). Macrovascular Complications of Type 2 Diabetes Mellitus. Curr. Vasc. Pharmacol..

[7] Faselis C., Katsimardou A., Imprialos K., Deligkaris P., Kallistratos M., Dimitriadis K. (2020). Microvascular Complications of Type 2 Diabetes Mellitus. Curr. Vasc. Pharmacol..

[8] Ogurtsova K., da Rocha Fernandes J.D., Huang Y., Linnenkamp U., Guariguata L., Cho N.H., Cavan D., Shaw J.E., Makaroff L.E. (2017). IDF Diabetes Atlas: Global estimates for the prevalence of diabetes for 2015 and 2040. Diabetes Res. Clin. Pract..

[9] American Diabetes Association (2013). Economic costs of diabetes in the U.S. in 2012. Diabetes Care.

[10] (2011). Emerging Risk Factors Collaboration. Diabetes mellitus, fasting glucose, and risk of cause-specific death. N. Engl. J. Med..

[11] Emerging Risk Factors Collaboration (2010). Diabetes mellitus, fasting blood glucose concentration, and risk of vascular disease: A collaborative meta-analysis of 102 prospective studies. Lancet.

[12] Tancredi M., Rosengren A., Svensson A.M., Kosiborod M., Pivodic A., Gudbjörnsdottir S., Wedel H., Clements M., Dahlqvist S., Lind M. (2015). Excess Mortality among Persons with Type 2 Diabetes. N. Engl. J. Med..

[13] Atchison E.A., Gridley G., Carreon J.D., Leitzmann M.F., McGlynn K.A. (2011). Risk of cancer in a large cohort of U.S. veterans with diabetes. Int. J. Cancer.

[14] El Sayed N.A., Aleppo G., Aroda V.R., Bannuru R.R., Brown F.M., Bruemmer D., Collins B.S., Hilliard M.E., Isaacs D., Johnson E.L. (2023). 11. Chronic Kidney Disease and Risk Management: Standards of Care in Diabetes—2023. Diabetes Care.

[15] Beam A., Clinger E., Hao L. (2021). Effect of Diet and Dietary Components on the Composition of the Gut Microbiota. Nutrients.

[16] Martínez-González M.A., Salas-Salvadó J., Estruch R., Corella D., Fitó M., Ros E., PREDIMED Investigators (2015). Benefits of the Mediterranean Diet: Insights from the PREDIMED Study. Prog. Cardiovasc. Dis..

[17] Fitó M., Guxens M., Corella D., Sáez G., Estruch R., de la Torre R., Francés F., Cabezas C., López-Sabater M.D., Marrugat J. (2007). Effect of a traditional Mediterranean diet on lipoprotein oxidation: A randomized controlled trial. Arch. Intern. Med..

[18] Esposito K., Maiorino M.I., Bellastella G., Panagiotakos D.B., Giugliano D. (2017). Mediterranean diet for type 2 diabetes: Cardiometabolic benefits. Endocrine.

[19] Schulze M.B., Schulz M., Heidemann C., Schienkiewitz A., Hoffmann K., Boeing H. (2007). Fiber and magnesium intake and incidence of type 2 diabetes: A prospective study and meta-analysis. Arch. Intern. Med..

[20] Hu F.B., van Dam R.M., Liu S. (2001). Diet and risk of Type II diabetes: The role of types of fat and carbohydrate. Diabetologia.

[21] Saulle R., La Torre G. (2010). The Mediterranean Diet, recognized by UNESCO as a cultural heritage of humanity. Ital. J. Public Health.

[22] Bendall C.L., Mayr H.L., Opie R.S., Bes-Rastrollo M., Itsiopoulos C., Thomas C.J. (2018). Central obesity and the Mediterranean diet: A systematic review of intervention trials. Crit. Rev. Food Sci. Nutr..

[23] International Diabetes Federation (2019). IDF Diabetes Atlas.

[24] Salas-Salvadó J., Guasch-Ferré M., Lee C.H., Estruch R., Clish C.B., Ros E. (2016). Protective Effects of the Mediterranean Diet on Type 2 Diabetes and Metabolic Syndrome. J. Nutr..

[25] American Diabetes Association Professional Practice Committee (2024). Facilitating Positive Health Behaviors and Well-being to Improve Health Outcomes: Standards of Care in Diabetes—2024. Diabetes Care.

[26] Milenkovic T., Bozhinovska N., Macut D., Bjekic-Macut J., Rahelic D., Velija Asimi Z., Burekovic A. (2021). Mediterranean Diet and Type 2 Diabetes Mellitus: A Perpetual Inspiration for the Scientific World. A Review. Nutrients.

[27] Tuomilehto J., Lindström J., Eriksson J.G., Valle T.T., Hämäläinen H., Ilanne-Parikka P., Keinänen-Kiukaanniemi S., Laakso M., Louheranta A., Rastas M. (2001). Prevention of type 2 diabetes mellitus by changes in lifestyle among subjects with impaired glucose tolerance. N. Engl. J. Med..

[28] Knowler W.C., Barrett-Connor E., Fowler S.E., Hamman R.F., Lachin J.M., Walker E.A., Nathan D.M. (2002). Diabetes Prevention Program Research Group. Reduction in the incidence of type 2 diabetes with lifestyle intervention or metformin. N. Engl. J. Med..

[29] Martínez-González M.A., de la Fuente-Arrillaga C., Nunez-Cordoba J.M., Basterra-Gortari F.J., Beunza J.J., Vazquez Z., Benito S., Tortosa A., Bes-Rastrollo M. (2008). Adherence to Mediterranean diet and risk of developing diabetes: Prospective cohort study. BMJ.

[30] Trichopoulou A., Costacou T., Bamia C., Trichopoulos D. (2003). Adherence to a Mediterranean diet and survival in a Greek population. N. Engl. J. Med..

[31] Martin-Moreno J.M., Boyle P., Gorgojo L., Maisonneuve P., Fernandez-Rodriguez J.C., Salvini S., Willett W.C. (1993). Development and validation of a food frequency questionnaire in Spain. Int. J. Epidemiol..

[32] Koloverou E., Esposito K., Giugliano D., Panagiotakos D. (2014). The effect of Mediterranean diet on the development of type 2 diabetes mellitus: A meta-analysis of 10 prospective studies and 136,846 participants. Metabolism.

[33] Esposito K., Maiorino M.I., Bellastella G., Chiodini P., Panagiotakos D., Giugliano D. (2015). A journey into a Mediterranean diet and type 2 diabetes: A systematic review with meta-analyses. BMJ Open.

[34] Toobert D.J., Glasgow R.E., Strycker L.A., Barrera M., Radcliffe J.L., C Wander R.C., Bagdade J.D. (2003). Biologic and quality-of-life outcomes from the Mediterranean Lifestyle Program: A randomized clinical trial. Diabetes Care.

[35] Elhayany A., Lustman A., Abel R., Attal-Singer J., Vinker S. (2010). A low-carbohydrate Mediterranean diet improves cardiovascular risk factors and diabetes control among overweight patients with type 2 diabetes mellitus: A 1-year prospective randomized intervention study. Diabetes Obes. Metab..

[36] Esposito K., Chiodini P., Maiorino M.I., Bellastella G., Panagiotakos D., Giugliano D. (2014). Which diet for prevention of type 2 diabetes? A meta-analysis of prospective studies. Endocrine.

[37] Esposito K., Marfella R., Ciotola M., Di Palo C., Giugliano F., Giugliano G., D’Armiento M., D’Andrea F., Giugliano D. (2004). Effect of a mediterranean-style diet on endothelial dysfunction and markers of vascular inflammation in the metabolic syndrome: A randomized trial. J. Am. Med. Assoc..

[38] Babio N., Toledo E., Estruch R., Ros E., Martínez-González M.A., Castañer O., Bulló M., Corella D., Arós F., Gómez-Gracia E. (2014). Mediterranean diets and metabolic syndrome status in the PREDIMED randomized trial. Can. Med. Assoc. J..

[39] Estruch R., Ros E., Salas-Salvadó J., Covas M.I., Corella D., Arós F., Gómez-Gracia E., Ruiz-Gutiérrez V., Fiol M., Lapetra J. (2018). Primary Prevention of Cardiovascular Disease with a Mediterranean Diet Supplemented with Extra-Virgin Olive Oil or Nuts. N. Engl. J. Med..

[40] Salas-Salvadó J., Bulló M., Babio N., Martínez-González M.Á., Ibarrola-Jurado N., Basora J., Estruch R., Covas M.I., Corella D., Arós F. (2011). Reduction in the incidence of type 2 diabetes with the Mediterranean diet: Results of the PREDIMED-Reus nutrition intervention randomized trial. Diabetes Care.

[41] Salas-Salvadó J., Bulló M., Estruch R., Ros E., Covas M.I., Ibarrola-Jurado N., Corella D., Arós F., Gómez-Gracia E., Ruiz-Gutiérrez V. (2014). Prevention of diabetes with Mediterranean diets: A subgroup analysis of a randomized trial. Ann. Intern. Med..

[42] Wallerer S., Stadelmaier J., Floegel E., Kiesswetter E., Bantle G., Hoffmann G., Schwingshackl L. (2025). Adherence to Mediterranean Diet and Risk of Type 2 Diabetes: An Updated Systematic Review and Dose-Response Meta-analysis. Adv. Nutr..

[43] Muscogiuri G., Maiorino M.I., Paolini B., Aversano F., Buscemi C., Cappiello I., Caruso I., Ceriani E., Chimienti M., Cicero F.A.G. (2026). Mediterranean diet for the primary prevention of cardiometabolic diseases: Evidence from a systematic review and meta-analysis featured in the Italian National Guidelines “La Dieta Mediterranea”. Nutrition.

[44] Ying Z., Fu M., Fang Z., Ye X., Wang P., Lu J. (2024). Mediterranean diet lowers risk of new-onset diabetes: A nationwide cohort study in China. Nutr. J..

[45] Sobiecki J.G., Imamura F., Davis C.R., Sharp S.Y., Koulman A., Hodgson J.M., Guevara M., Schulze M.B., Zheng J.S., Agnoli C. (2023). A nutritional biomarker score of the Mediterranean diet and incident type 2 diabetes: Integrated analysis of data from the MedLey randomised controlled trial and the EPIC-InterAct case-cohort study. PLoS Med..

[46] Schwingshackl L., Missbach B., König J., Hoffmann G. (2015). Adherence to a Mediterranean diet and risk of diabetes: A systematic review and meta-analysis. Public Health Nutr..

[47] Jannasch F., Kröger J., Schulze M.B. (2017). Dietary Patterns and Type 2 Diabetes: A Systematic Literature Review and Meta-Analysis of Prospective Studies. J. Nutr..

[48] Zheng M., Hu P., Yang S., Wang J., Chen R., Liu Y., Chen M., Sun S. (2018). A review of the effects of Mediterranean diet on prevention of type 2 diabetes amongst overweight patients. IOP Conf. Ser. Mater. Sci. Eng..

[49] Sarsangi P., Salehi-Abargouei A., Ebrahimpour-Koujan S., Esmaillzadeh A. (2022). Association between Adherence to the Mediterranean Diet and Risk of Type 2 Diabetes: An Updated Systematic Review and Dose-Response Meta-Analysis of Prospective Cohort Studies. Adv. Nutr..

[50] Zeraattalab-Motlagh S., Jayedi A., Shab-Bidar S. (2022). Mediterranean dietary pattern and the risk of type 2 diabetes: A systematic review and dose-response meta-analysis of prospective cohort studies. Eur. J. Nutr..

[51] Martinez-Lacoba R., Pardo-Garcia I., Amo-Saus E., Escribano-Sotos F. (2018). Mediterranean diet and health outcomes: A systematic meta-review. Eur. J. Public Health.

[52] Ajala O., English P., Pinkney J. (2013). Systematic review and meta-analysis of different dietary approaches to the management of type 2 diabetes. Am. J. Clin. Nutr..

[53] Huo R., Du T., Xu Y., Xu W., Chen X., Sun K., Yu X. (2015). Effects of Mediterranean-style diet on glycemic control, weight loss and cardiovascular risk factors among type 2 diabetes individuals: A meta-analysis. Eur. J. Clin. Nutr..

[54] Carter P., Achana F., Troughton J., Gray L.J., Khunti K., Davies M.J. (2014). A Mediterranean diet improves HbA1c but not fasting blood glucose compared to alternative dietary strategies: A network meta-analysis. J. Hum. Nutr. Diet..

[55] Nordmann A.J., Suter-Zimmermann K., Bucher H.C., Shai I., Tuttle K.R., Estruch R., Briel M. (2011). Meta-analysis comparing Mediterranean to low-fat diets for modification of cardiovascular risk factors. Am. J. Med..

[56] Rees K., Hartley L., Flowers N., Clarke A., Hooper L., Thorogood M., Stranges S. (2013). ‘Mediterranean’ dietary pattern for the primary prevention of cardiovascular disease. Cochrane Database Syst. Rev..

[57] Esposito K., Kastorini C.-M., Panagiotakos D.B., Giugliano D. (2011). Mediterranean diet and weight loss: Meta-analysis of randomized controlled trials. Metab. Syndr. Relat. Disord..

[58] Panagiotakos D.B., Tzima N., Pitsavos C., Chrysohoou C., Zampelas A., Toussoulis D., Stefanadis C. (2007). The association between adherence to the Mediterranean diet and fasting indices of glucose homeostasis: The ATTICA Study. J. Am. Coll. Nutr..

[59] Emadian A., Andrews R.C., England C.Y., Wallace V., Thompson J.L. (2015). The effect of macronutrients on glycaemic control: A systematic review of dietary randomised controlled trials in overweight and obese adults with type 2 diabetes in which there was no difference in weight loss between treatment groups. Br. J. Nutr..

[60] Schwingshackl L., Chaimani A., Hoffmann G., Schwedhelm C., Boeing H. (2018). A network meta-analysis on the comparative efficacy of different dietary approaches on glycaemic control in patients with type 2 diabetes mellitus. Eur. J. Epidemiol..

[61] Becerra-Tomás N., Blanco Mejía S., Viguiliouk E., Khan T., Kendall C.W.C., Kahleova H., Rahelić D., Sievenpiper J.L., Salas-Salvadó J. (2020). Mediterranean diet, cardiovascular disease and mortality in diabetes: A systematic review and meta-analysis of prospective cohort studies and randomized clinical trials. Crit. Rev. Food Sci. Nutr..

[62] Wu L., Parhofer K.G. (2014). Diabetic dyslipidemia. Metabolism.

[63] Whiteley C., Benton F., Matwiejczyk L., Luscombe-Marsh N. (2023). Determining Dietary Patterns to Recommend for Type 2 Diabetes: An Umbrella Review. Nutrients.

[64] Zheng X., Zhang W., Wan X., Lv X., Lin P., Si S., Xue F., Wang A., Cao Y. (2024). The effects of Mediterranean diet on cardiovascular risk factors, glycemic control and weight loss in patients with type 2 diabetes: A meta-analysis. BMC Nutr..

[65] Pan B., Wu Y., Yang Q., Ge L., Gao C., Xun Y., Tian J., Ding G. (2019). The impact of major dietary patterns on glycemic control, cardiovascular risk factors, and weight loss in patients with type 2 diabetes: A network meta-analysis. J. Evid. Based Med..

[66] Wu M.-J., Hung C.-H., Yong S.-B., Ching G.S., Hsu H.-J. (2025). Impact of the Mediterranean Diet on Glycemic Control, Body Mass Index, Lipid Profile, and Blood Pressure in Type 2 Diabetes: A Meta-Analysis of Randomized Controlled Trials. Nutrients.

[67] Lauria F., Formisano A., Dello Russo M., Quaglia C., Giacco R., Russo G.L., Spagnuolo C., Vitale M. (2026). Mediterranean diet, gut microbiota, and type 2 diabetes: A systematic review and meta-analysis of intervention trials. Nutr. Metab. Cardiovasc. Dis..

[68] DeFronzo R.A., Ferrannini E., Groop L., Henry R.R., Herman W.H., Holst J.J., Hu F.B., Kahn C.R., Raz I., Shulman G.I. (2015). Type 2 diabetes mellitus. Nat. Rev. Dis. Prim..

[69] DeFronzo R.A. (2010). Insulin resistance, lipotoxicity, type 2 diabetes and atherosclerosis: The missing links. The Claude Bernard Lecture 2009. Diabetologia.

[70] Bensellam M., Laybutt D.R., Jonas J.C. (2012). The molecular mechanisms of pancreatic β-cell glucotoxicity: Recent findings and future research directions. Mol. Cell. Endocrinol..

[71] Collins S., Pi J., Yehuda-Shnaidman E. (2012). Uncoupling and reactive oxygen species (ROS)—A double-edged sword for β-cell function? “Moderation in all things”. Best Pract. Res. Clin. Endocrinol. Metab..

[72] DeFronzo R.A. (2009). Banting Lecture. From the triumvirate to the ominous octet: A new paradigm for the treatment of type 2 diabetes mellitus. Diabetes.

[73] Itsiopoulos C., Brazionis L., Kaimakamis M., Cameron M., Best J.D., O’Dea K., Rowley K. (2011). Can the Mediterranean diet lower HbA1c in type 2 diabetes? Results from a randomized cross-over study. Nutr. Metab. Cardiovasc. Dis..

[74] Maiorino M.I., Bellastella G., Petrizzo M., Scappaticcio L., Giugliano D., Esposito K. (2016). Mediterranean diet cools down the inflammatory milieu in type 2 diabetes: The MÉDITA randomized controlled trial. Endocrine.

[75] Martín-Peláez S., Fito M., Castaner O. (2020). Mediterranean Diet Effects on Type 2 Diabetes Prevention, Disease Progression, and Related Mechanisms: A Review. Nutrients.

[76] Eid H.M., Martineau L.C., Saleem A., Muhammad A., Vallerand D., Benhaddou-Andaloussi A., Nistor L., Afshar A., Arnason J.T., Haddad P.S. (2010). Stimulation of AMP-activated protein kinase and enhancement of basal glucose uptake in muscle cells by quercetin and quercetin glycosides, active principles of the antidiabetic medicinal plant *Vaccinium vitis-idaea*. Mol. Nutr. Food Res..

[77] Dhanya R., Arya A.D., Nisha P., Jayamurthy P. (2017). Quercetin, a Lead Compound against Type 2 Diabetes Ameliorates Glucose Uptake via AMPK Pathway in Skeletal Muscle Cell Line. Front. Pharmacol..

[78] Torres-Peña J.D., Garcia-Rios A., Delgado-Casado N., Gomez-Luna P., Alcala-Diaz J.F., Yubero-Serrano E.M., Gomez-Delgado F., Leon-Acuña A., Lopez-Moreno J., Camargo A. (2018). Mediterranean diet improves endothelial function in patients with diabetes and prediabetes: A report from the CORDIOPREV study. Atherosclerosis.

[79] Berger M.M., Delodder F., Liaudet L., Tozzi P., Schlaepfer J., Chiolero R.L., Tappy L. (2013). Three short perioperative infusions of n-3 PUFAs reduce systemic inflammation induced by cardiopulmonary bypass surgery: A randomized controlled trial. Am. J. Clin. Nutr..

[80] Maedler K., Oberholzer J., Bucher P., Spinas G.A., Donath M.Y. (2003). Monounsaturated fatty acids prevent the deleterious effects of palmitate and high glucose on human pancreatic beta-cell turnover and function. Diabetes.

[81] Carpentier Y.A., Portois L., Malaisse W.J. (2006). n-3 fatty acids and the metabolic syndrome. Am. J. Clin. Nutr..

[82] Risérus U., Willett W.C., Hu F.B. (2009). Dietary fats and prevention of type 2 diabetes. Prog. Lipid Res..

[83] Visioli F., Poli A., Gall C. (2002). Antioxidant and other biological activities of phenols from olives and olive oil. Med. Res. Rev..

[84] Nie C., He T., Zhang W., Zhang G., Ma X. (2018). Branched Chain Amino Acids: Beyond Nutrition Metabolism. Int. J. Mol. Sci..

[85] Lynch C.J., Adams S.H. (2014). Branched-chain amino acids in metabolic signalling and insulin resistance. Nat. Rev. Endocrinol..

[86] Zhenyukh O., Civantos E., Ruiz-Ortega M., Sánchez M.S., Vázquez C., Peiró C., Egido J., Mas S. (2017). High concentration of branched-chain amino acids promotes oxidative stress, inflammation and migration of human peripheral blood mononuclear cells via mTORC1 activation. Free Radic. Biol. Med..

[87] Ruiz-Canela M., Guasch-Ferré M., Toledo E., Clish C.B., Razquin C., Liang L., Wang D.D., Corella D., Estruch R., Hernáez Á. (2018). Plasma branched chain/aromatic amino acids, enriched Mediterranean diet and risk of type 2 diabetes: Case-cohort study within the PREDIMED Trial. Diabetologia.

[88] Nicholson J.K., Holmes E., Kinross J., Burcelin R., Gibson G., Jia W., Pettersson S. (2012). Host-gut microbiota metabolic interactions. Science.

[89] Blandino G., Inturri R., Lazzara F., Di Rosa M., Malaguarnera L. (2016). Impact of gut microbiota on diabetes mellitus. Diabetes Metab..

[90] Zmora N., Suez J., Elinav E. (2019). You are what you eat: Diet, health and the gut microbiota. Nat. Rev. Gastroenterol. Hepatol..

[91] Larsen N., Vogensen F.K., van den Berg F.W., Nielsen D.S., Andreasen A.S., Pedersen B.K., Al-Soud W.A., Sørensen S.J., Hansen L.H., Jakobsen M. (2010). Gut microbiota in human adults with type 2 diabetes differs from non-diabetic adults. PLoS ONE.

[92] Karlsson F.H., Tremaroli V., Nookaew I., Bergström G., Behre C.J., Fagerberg B., Nielsen J., Bäckhed F. (2013). Gut metagenome in European women with normal, impaired and diabetic glucose control. Nature.

[93] Sato J., Kanazawa A., Ikeda F., Yoshihara T., Goto H., Abe H., Komiya K., Kawaguchi M., Shimizu T., Ogihara T. (2014). Gut dysbiosis and detection of “live gut bacteria” in blood of Japanese patients with type 2 diabetes. Diabetes Care.

[94] Qin J., Li Y., Cai Z., Li S., Zhu J., Zhang F., Liang S., Zhang W., Guan Y., Shen D. (2012). A metagenome-wide association study of gut microbiota in type 2 diabetes. Nature.

[95] Krznarić Ž., Vranešić Bender D., Meštrović T. (2019). The Mediterranean diet and its association with selected gut bacteria. Curr. Opin. Clin. Nutr. Metab. Care.

[96] Pedersen H.K., Gudmundsdottir V., Nielsen H.B., Hyotylainen T., Nielsen T., Jensen B.A., Forslund K., Hildebrand F., Prifti E., Falony G. (2016). Human gut microbes impact host serum metabolome and insulin sensitivity. Nature.

[97] Mandaliya D.K., Seshadri S. (2019). Short Chain Fatty Acids, pancreatic dysfunction and type 2 diabetes. Pancreatology.

[98] Puddu A., Sanguineti R., Montecucco F., Viviani G.L. (2014). Evidence for the gut microbiota short-chain fatty acids as key pathophysiological molecules improving diabetes. Mediat. Inflamm..

[99] Cani P.D., Delzenne N.M. (2009). The role of the gut microbiota in energy metabolism and metabolic disease. Curr. Pharm. Des..

[100] Zhao L., Zhang F., Ding X., Wu G., Lam Y.Y., Wang X., Fu H., Xue X., Lu C., Ma J. (2018). Gut bacteria selectively promoted by dietary fibers alleviate type 2 diabetes. Science.

[101] Salleh S.N., Fairus A.A.H., Zahary M.N., Bhaskar Raj N., Mhd Jalil A.M. (2019). Unravelling the Effects of Soluble Dietary Fibre Supplementation on Energy Intake and Perceived Satiety in Healthy Adults: Evidence from Systematic Review and Meta-Analysis of Randomised-Controlled Trials. Foods.

[102] Alahmari L.A. (2024). Dietary fiber influence on overall health, with an emphasis on CVD, diabetes, obesity, colon cancer, and inflammation. Front. Nutr..

[103] Tejani V.N., Dhillon S.S., Damarlapally N., Usman N.U.B., Winson T., Basu Roy P., Panjiyar B.K. (2023). The Relationship Between Dietary Fiber Intake and Blood Pressure Worldwide: A Systematic Review. Cureus.

[104] Kim M.H., Choi M.K. (2013). Seven dietary minerals (Ca, P, Mg, Fe, Zn, Cu, and Mn) and their relationship with blood pressure and blood lipids in healthy adults with self-selected diet. Biol. Trace Elem. Res..

[105] Dong J.-Y., Xun P., He K., Qin L.-Q. (2011). Magnesium intake and risk of type 2 diabetes: Meta-analysis of prospective cohort studies. Diabetes Care.

[106] Prasad M., Jayaraman S., Eladl M.A., El-Sherbiny M., Abdelrahman M.A.E., Veeraraghavan V.P., Vengadassalapathy S., Umapathy V.R., Jaffer Hussain S.F., Krishnamoorthy K. (2022). A Comprehensive Review on Therapeutic Perspectives of Phytosterols in Insulin Resistance: A Mechanistic Approach. Molecules.

[107] Escurriol V., Cofán M., Serra M., Bulló M., Basora J., Salas-Salvadó J., Corella D., Zazpe I., Martínez-González M.A., Ruiz-Gutiérrez V. (2009). Serum sterol responses to increasing plant sterol intake from natural foods in the Mediterranean diet. Eur. J. Nutr..

[108] Demonty I., Ras R.T., van der Knaap H.C., Meijer L., Zock P.L., Geleijnse J.M., Trautwein E.A. (2013). The effect of plant sterols on serum triglyceride concentrations is dependent on baseline concentrations: A pooled analysis of 12 randomised controlled trials. Eur. J. Nutr..

[109] Baliunas D.O., Taylor B.J., Irving H., Roerecke M., Patra J., Mohapatra S., Rehm J. (2009). Alcohol as a risk factor for type 2 diabetes: A systematic review and meta-analysis. Diabetes Care.

[110] Covas M.I., Gambert P., Fitó M., de la Torre R. (2010). Wine and oxidative stress: Up-to-date evidence of the effects of moderate wine consumption on oxidative damage in humans. Atherosclerosis.

[111] DiMauro A., Tuccinardi D., Watanabe M., Del Toro R., Monte L., Giorgino R., Rampa L., Rossini G., Kyanvash S., Soare-et A. (2021). The Mediterranean diet increases glucagon-like peptide 1 and oxyntomodulin compared with a vegetarian diet in patients with type 2 diabetes: A randomized controlled cross-over trial. Diabetes Metab. Res. Rev..

[112] Pocai A. (2013). Action and therapeutic potential of oxyntomodulin. Mol. Metab..

[113] García-Gorrita C., San Onofre N., Merino-Torres J.F., Soriano J.M. (2025). Beyond GLP-1 Agonists: An Adaptive Ketogenic–Mediterranean Protocol to Counter Metabolic Adaptation in Obesity Management. Nutrients.

[114] Barrea L., Annunziata G., Verde L., Galasso M., Savastano S., Colao A., Muscogiuri G. (2025). A Multidisciplinary Perspective on Semaglutide Treatment and Medical Nutrition Therapy in Obesity Management. Curr. Obes. Rep..

[115] Paternò V., Geraci G., Piticchio T., Le Moli R., Burgio S., Costanzo G., Sambataro G., Baratta R., Barbagallo F., Pallotti F. (2026). Mediterranean diet adherence and tirzepatide: Real-world evidence on adiposity indices and insulin resistance beyond weight loss. Front. Endocrinol..

[116] Podadera-Herreros A., Arenas-de Larriva A.P., Gutierrez-Mariscal F.M., Alcala-Diaz J.F., Ojeda-Rodriguez A., Rodriguez-Cantalejo F., Cardelo M.P., Rodriguez-Cano D., Torres-Peña J.D., Luque R.M. (2024). Mediterranean diet as a strategy for preserving kidney function in patients with coronary heart disease with type 2 diabetes and obesity: A secondary analysis of CORDIOPREV randomized controlled trial. Nutr. Diabetes.

[117] Pereira M.J., Eriksson J.W. (2019). Emerging Role of SGLT-2 Inhibitors for the Treatment of Obesity. Drugs.

[118] Hoffman R., Gerber M. (2013). Evaluating and adapting the Mediterranean diet for non-Mediterranean populations: A critical appraisal. Nutr. Rev..

[119] Woodside J., Young I.S., McKinley M.C. (2022). Culturally adapting the Mediterranean Diet pattern—A way of promoting more ‘sustainable’ dietary change?. Br. J. Nutr..

[120] Perrone P., Landriani L., Patalano R., Meccariello R., D’Angelo S. (2025). The Mediterranean Diet as a Model of Sustainability: Evidence-Based Insights into Health, Environment, and Culture. Int. J. Environ. Res. Public Health.

